# The pyroptosis-related signature predicts prognosis and influences the tumor immune microenvironment in dedifferentiated liposarcoma

**DOI:** 10.1515/med-2023-0886

**Published:** 2024-01-09

**Authors:** Wenjing Chen, Jun Cheng, Yiqi Cai, Pengfei Wang, Jinji Jin

**Affiliations:** Departments of Gastrointestinal Surgery, The First Affiliated Hospital of Wenzhou Medical University, Ouhai District, Wenzhou, 325003, Zhejiang Province, China

**Keywords:** pyroptosis, dedifferentiated liposarcoma, immune infiltrates, tumor microenvironment, immunotherapy

## Abstract

**Background:**

Dedifferentiated liposarcoma (DDL), a member of malignant mesenchymal tumors, has a high local recurrence rate and poor prognosis. Pyroptosis, a newly discovered programmed cell death, is tightly connected with the progression and outcome of tumor.

**Objective:**

The aim of this study was to explore the role of pyroptosis in DDL.

**Methods:**

We obtained the RNA sequencing data from The Cancer Genome Atlas (TCGA) and Genotype-Tissue Expression databases to identify different pyroptosis-related genes (PRGs) expression pattern. An unsupervised method for clustering based on PRGs was performed. Based on the result of cluster analysis, we researched clinical outcomes and immune microenvironment between clusters. The differentially expressed genes (DEGs) between the two clusters were used to develop a prognosis model by the LASSO Cox regression method, followed by the performance of functional enrichment analysis and single-sample gene set enrichment analysis. All of the above results were validated in the Gene Expression Omnibus (GEO) dataset.

**Results:**

Forty-one differentially expressed PRGs were found between tumor and normal tissues. A consensus clustering analysis based on PRGs was conducted and classified DDL patients into two clusters. Cluster 2 showed a better outcome, higher immune scores, higher immune cells abundances, and higher expression levels in numerous immune checkpoints. DEGs between clusters were identified. A total of 5 gene signatures was built based on the DEGs and divided all DDL patients of the TCGA cohort into low-risk and high-risk groups. The low-risk group indicates greater inflammatory cell infiltration and better outcome. For external validation, the survival difference and immune landscape between the two risk groups of the GEO cohort were also significant. Receiver operating characteristic curves implied that the risk model could exert its function as an outstanding predictor in predicting DDL patients’ prognoses.

**Conclusion:**

Our findings revealed the clinical implication and key role in tumor immunity of PRGs in DDL. The risk model is a promising predictive tool that could provide a fundamental basis for future studies and individualized immunotherapy.

## 1 Introduction

Soft tissue sarcomas (STSs) are a series of rare tumors composed of more than 70 histological subtypes [1], accounting for almost 1% of all diagnosed cancers [2]. Liposarcoma, constituting approximately 20% of STSs, is the most common type [3]. Furthermore, the largest subgroup of liposarcoma is divided into dedifferentiated liposarcoma (DDL) and well-differentiated liposarcoma (WDL) [4]. Unlike WDL, DDL behaves more aggressively, with significantly higher local recurrence rates (40%), higher metastatic rate (15–30%), and poorer prognosis [5]. DDL is characterized by a high-level amplified chromosomal region 12q13–15, including amplification of MDM2 and CDK4. Retroperitoneum is the most common location of DDL, while it can also occur in extremities and trunk [6]. Within the past decade, systemic treatments including chemotherapy and molecular targeted agents become all the rage, but surgical resection remains the fundamental method of curing DDL [7], whose recurrence and prognosis have not been markedly improved.

Pyroptosis is a highly specific type of inflammatory cell death that is largely dependent on Gasdermin family [8]. Caspase-1 and caspase-4/5/11 cleave gasdermins, which then lead to cell swelling, lysis, and release inflammatory cytokines, thus triggering a strong inflammatory response [9,10,11]. Moderate pyroptosis is conducive to maintaining a stable intracellular environment and effectively preventing excessive proliferation of cells [12]. Pyroptosis also plays a crucial role in multiple tumors. GSDMD, of which the expression was decreased in gastric cancer (GC) cells compared with adjacent normal cells, acts as a tumor suppressor [13]. However, GSDMD can induce invasion and tumor progression in lung cancer [14]. The same dual roles were also observed in other Gasdermin proteins, including GSDME, a molecule that is extensively studied in pyroptosis [15]. The role of pyroptosis in cancer may act as a double-edged sword, as pyroptosis creating an environment that inhibits tumor growth while accelerating tumor growth by weakening the immunity of patients [16,17]. Pyroptosis can also regulate malignant phenotypes and chemotherapy resistance, affecting tumor progression and patients’ prognosis [18]. Several previous studies have confirmed that GSDME-mediated pyroptosis can promote the toxin side effects of chemotherapy [19,20]. Furthermore, GSDME switches caspase-3-dependent apoptosis induced by 5-FU into pyroptosis in GC cells [21].

Although pyroptosis has been observed in a variety of tumors, its potential role in DDL is poorly understood. STSs are deemed to a group of various subtypes, and the treatment and prognosis of each type were different. In clinical work, we have observed that the prognosis of DDL is extremely poor [22], so we hope to investigate the role of pyroptosis in DDL and its impact on patient outcomes and establish a prognostic model related to pyroptosis in DDL to provide help for clinical work and further research. In the present study, with DDL patients divided into two pyroptosis-related subtypes, the prognosis and immune infiltration differed in terms of subtypes. We further established a scoring system through the LASSO-Cox method. This scoring system can be a convincing way to predict prognosis and immune infiltration. Our research marked the dawn of potential association between pyroptosis, the immune microenvironment, and outcomes of DDL patients.

## 2 Methods

### 2.1 Data acquisition

RNA sequencing profile of 56 DDL patients of TCGA-SARC and 515 adipose tissues of Genotype-Tissue Expressions cohort were downloaded from the UCSC browser (https://xenabrowser.net) on 12 October 2021. The corresponding clinical data of DDL patients were extracted from the cBioPortal (http://www.cbioportal.org/) (Table 1). The somatic mutation information and copy number variation (CNV) information of DDL were downloaded from the cancer genome atlas (TCGA) dataset. The “maftools” package was applied to present the mutation landscape by waterfall plots and calculate the CNV frequency of pyroptosis-related genes (PRGs) by lollipop charts. The location of PRGs with CNV information on chromosomes was presented by the “RCircos” package. We also obtained the gene expression profiling and corresponding clinical information of GSE30929 from the Gene Expression Omnibus (GEO) database (https://www.ncbi.nlm.nih.gov/geo/) as a validation cohort, which includes totally 40 DDL patients.

**Table 1:** Characteristics of DDL patients

Characteristic	Overall (*N* = 56)
**Age**	
>65	25 (44.64%)
≤65	31 (55.36%)
**Gender**	
Female	19 (33.93%)
Male	37 (66.07%)
**Surgical margin**	
R0	24 (42.86%)
≥R1	32 (57.14%)
**Tumor tissue site**	
Chest	1 (1.79%)
Intraabdominal	5 (8.93%)
Lower extremity	3 (5.36%)
Pelvic	4 (7.14%)
Retroperitoneum	42 (75.00%)
Upper extremity	1 (1.79%)

### 2.2 Construction of pyroptosis-related clusters

We collected totally 52 PRGs those are proved by previous studies [18,^23^]. The “limma” package was used to identify different expression of PRGs between DDL and adipose tissues. A PPI network for the PRGS was constructed by the online tools on the STRING website (http://string-db.org). We used eight algorithms, namely Bottleneck, EPC, Degree, Cloness, Maximal Clique Centrality, Maximum Neighborhood Component, Density of Maximum Neighborhood Component, and EcCentricity, to calculate the top 10 hub genes using cytoscape. Then, we used “ConsensusClusterPlus” R package to identify distinct patterns based on PRGs by the k-means method. We repeated this process 50 times to ensure our classification’s stability. Then, we also used the “limma” package to obtain differentially expressed genes (DEGs) by comparing the different clusters.

### 2.3 Gene set enrichment analysis (GSEA)

GSEA was performed using the Java GSEA desktop application. In this study, GSEA v4.1.0 software was used to identify immunological features in clusters. Nominal (NOM) *p* value <0.05 and false discovery rate <0.05 were considered significant enrichment.

### 2.4 Gene set variation analysis (GSVA)

GSVA enrichment analysis was performed in heatmap by the use of “GSVA” R packages [24]. “c2.cp.kegg.v7.4.symbols.gmt” and “h.all.v7.4.symbols.gmt” were downloaded from MSigDB. Differences were believed as statistically significant if adjusted *p*-values <0.05.

### 2.5 Immune correlation analysis

We used the CIBERSORT algorithm to evaluate 22 human immune cell subsets of each group sample. R software package “ESTIMATE” was used to assess the “Immunescore,” “Stromalscore,” and “Estimatescore” [25]. Single-sample gene-set enrichment analysis (ssGSEA) was used to determine the levels of immune cell infiltration in different clusters and risk groups. Furthermore, Quantiseq, Timer, Mcp_counter, and Xcell [26,27] were also used to calculate the prevalence of immune cells in different cluster samples. In addition, we collected information about 122 immunomodulators, including Chemokines, Receptors, MHC, Immunoinhibitor, and Immunostimulants [28,29]. Cancer immunity and anti-cancer immune system response were reflected by analyzing the differential expression of immunomodulators in different clusters.

We also extracted 23 N6-methyladenosine (m6A) regulatory genes (METTL3, METTL14, METTL16, WTAP, VIRMA, ZC3H13, RBM15, RBM15B, YTHDC1, YTHDC2, YTHDF1, YTHDF2, YTHDF3, HNRNPC, FMR1, LRPPRC, HNRNPA2B1, IGFBP1, IGFBP2, IGFBP3, RBMX, FTO, ALKBH5) from previous studies [30,31] and compared the different expression levels of these genes in clusters.

### 2.6 The establishment and validation of the prognostic gene model

In the TCGA cohort, we established an efficient prediction model using LASSO-Cox analysis. Five survival-related genes were used to construct the model, calculating the risk score for each patient. Risk score = (Exp gene1 × coefficient gene1) + (Exp gene2 × coefficient gene2) + ⋯ + (Exp gene5 × coefficient gene5). Fifty-six DDL patients were divided into high- and low-risk groups according to the median risk score, and the Kaplan–Meier analysis of overall survival (OS) time, 1-, 3-, and 5-year receiver operating characteristic (ROC) curve, principal component analysis (PCA), and t-SNE was performed. Following the same procedure, GSE30929 was used as a testing dataset to calculate the patient’s risk score for external validation.

### 2.7 Independent prognostic analysis of the risk score

We obtained the DDL patients’ clinical data (age, gender, Surgical Margin) of the TCGA cohort from cBioPortal. These clinical data together with the risk score were analyzed through the univariate and multivariable Cox regression models. Cox regression model was not analyzed in the GEO cohort due to the lack of clinical data.

### 2.8 Statistical analysis

All statistical analyses were performed by R software version 3.6.3. Differences between two groups were assessed by using the Wilcox test. Kaplan–Meier method with a two-sided log-rank test was used to compare the OS of patients between subgroups. LASSO Cox regression analysis was derived by using the “glmnet” packages. Time-dependent ROC curves and the area under curves (AUC) were derived by using the “timeROC” packages. *p* < 0.05 was considered statistically significant.


**Ethics approval and consent to participate:** Not applicable.
**Human and animal rights:** No animals/humans were used for studies that are the basis of this research.

## 3 Results

### 3.1 Expression variation and genetic changes of PRGs in DDL

**Figure 1:**
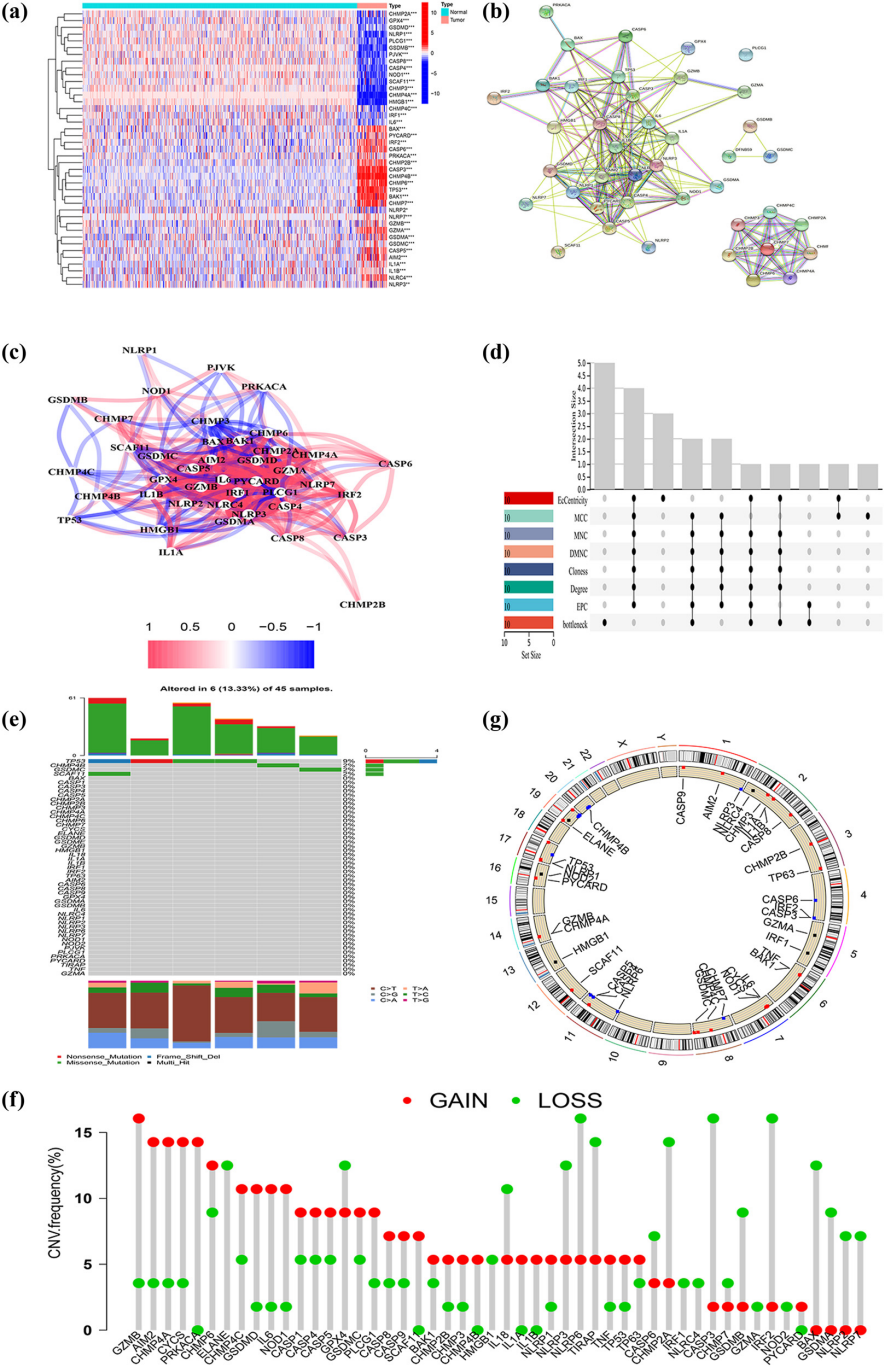
Overview of genetic and expression variation of PRGs in DDL. (a) Heatmap of the PRGs between the normal and the tumor tissues. (b) PPI network showing the interactions of the PRGs. (c) The correlation network of the PRGs (red line: positive correlation; blue line: negative correlation). (d) UpSet plot shows the hub genes from the eight algorithms of cytohubba. (e) The mutation frequency of PRGs in DDL patients from the TCGA dataset. Numbers on the right represent frequencies. Different colors in the bottom annotation represent different mutation types. (f) Frequencies of CNV gain, loss, and non-CNV among PRGs. (g) Locations of CNV alterations in PRGs on 23 chromosomes. *p < 0.05; **p < 0.01; ***p < 0.001.

This study included a total of 52 PRGs, and we found that 41 PRGs were expressed significantly different between cancer and normal tissues (*p* < 0.005) (Figure 1a). Among them, 18 genes (CHMP4A, GSDMB, PJVK, IL6, CHMP4C, NLRP1, CASP4, NOD1, HMGB1, CASP8, PLCG1, IRF1, CHMP3, SCAF11, GSDMD, GPX4, NLRP2, CHMP2A) were downregulated and 23 genes (PRKACA, PYCARD, BAX, IRF2, CHMP2B, CHMP7, IL1B, NLRP3, CASP6, CHMP4B, CHMP6, TP53, GZMB, GSDMC, CASP3, BAK1, NLRC4, GSDMA, GZMA, IL1A, CASP5, AIM2, NLRP7) were upregulated, we also constructed the PPI network and correlation network containing those different expressed PRGs (Figure 1b and c). Next, we identified hub genes by using the cytoHubba plugin. Ten hub genes (IL1A, IL1B, IL6, CASP3, CASP5, CASP8, AIM2, PYCARD, NLRC4, NLRP) were identified by intersecting the results from the eight algorithms of cytohubba (Figure 1d).

At the genetic level, 6 of the 45 DDL samples (about 13.33%) showed PRG mutations, and TP53 showing the highest frequency (9%), followed by CHMP4B and GSDMC (Figure 1e). Then, we explored somatic copy number alterations in these PRGs and found that PRGs displayed prevalent CNV alterations (Figure 1f). The locations of the CNV alterations on chromosomes are shown in Figure 1g.

### 3.2 Immune infiltration analyses between DDL and controls

**Figure 2:**
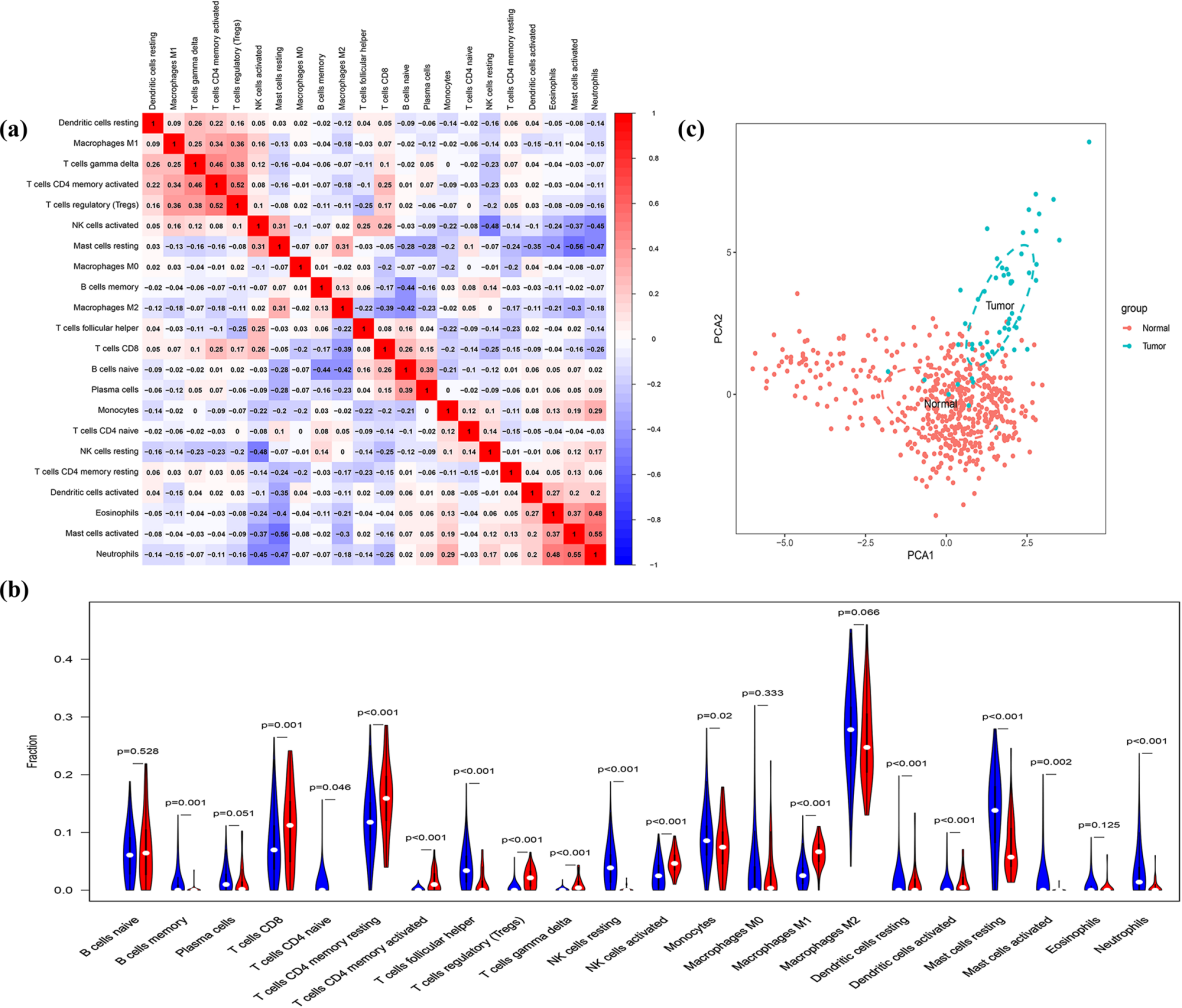
Results of CIBERSORT analysis and immune infiltration between DDL and adipose groups. (a) Correlation matrix of infiltration degree of immune cells in DDL samples. Red indicates trends consistent with the positive correlation and blue indicates trends consistent with the negative correlation between two immune cells. The bigger size of the numbers statistics data represents the more positive or negative correlation. (b) Violin diagram of immune cell proportions in two groups. The blue fusiform fractions on the left represent the adipose group and the red fusiform fractions on the right represent the DDL group. (c) PCA was performed in two groups.

CIBERSORT was applied to explore the features of totally 22 types of immune cells’ distribution in DDL samples. The corheatmap (Figure 2a) result showed that Neutrophils and Mast cells activated had a positive correlation (value = 0.55). Mast cells resting had a negative correlation with Mast cells activated (value = −0.56). Except for B cells naive, plasma cells, macrophage M0, macrophage M2, and eosinophil immune cells, the remaining 17 immune cells are infiltrated statistically less or more in DDL compared with the adipose group (Figure 2b). PCA depicted no overlap of these two elliptical clusters in immune cell infiltration between the DDL and control groups (Figure 2c).

### 3.3 PRG-related clusters identified in DDL

**Figure 3:**
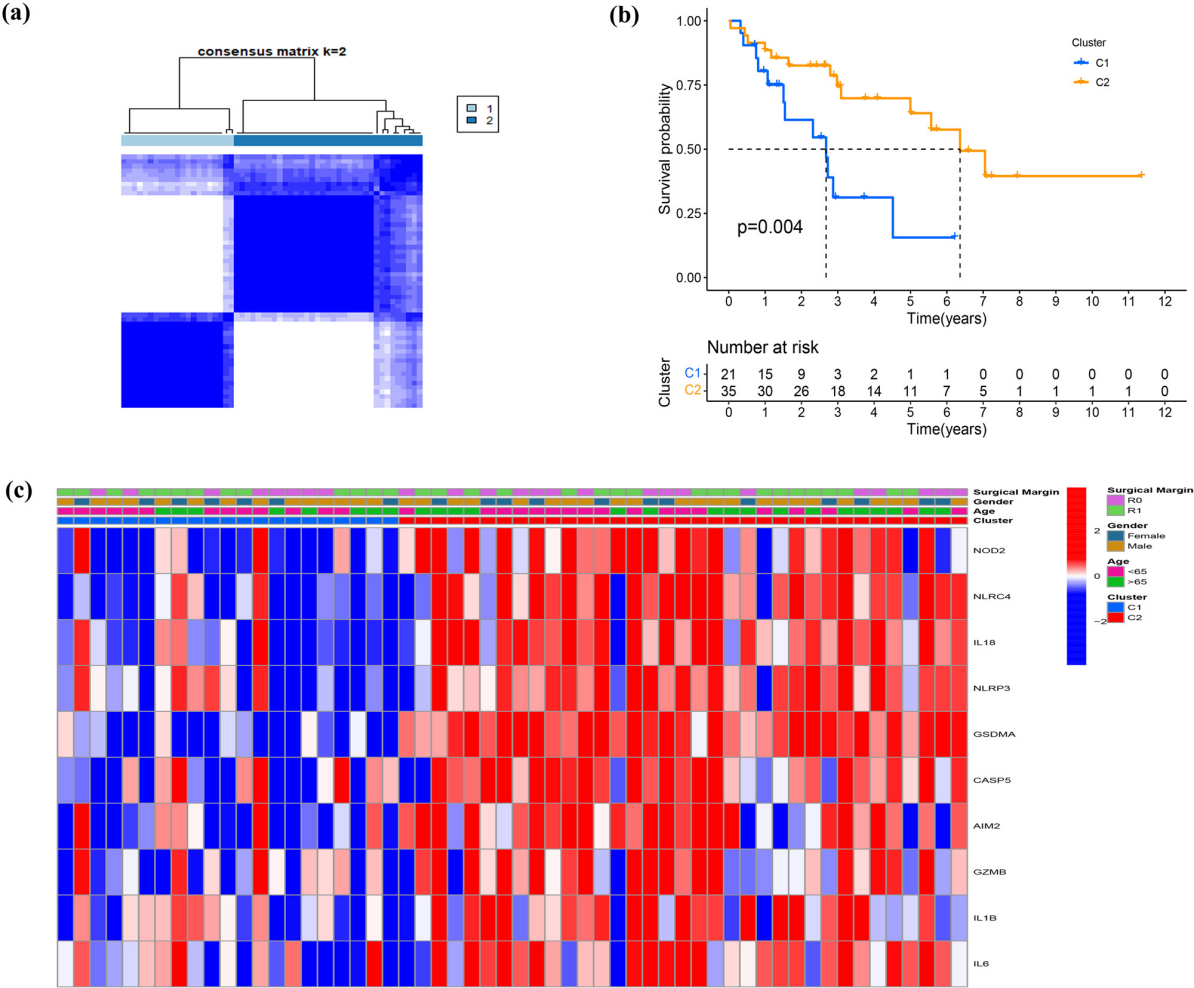
Tumor classification based on the PRGs. (a) DDL patients were divided into two clusters by the consensus clustering matrix (*k* = 2). (b) Kaplan–Meier curves for the OS of these two clusters. (c) Heatmap and the clinicopathologic characters of the two clusters classified by these differentially expressed PRGs.

**Figure 4:**
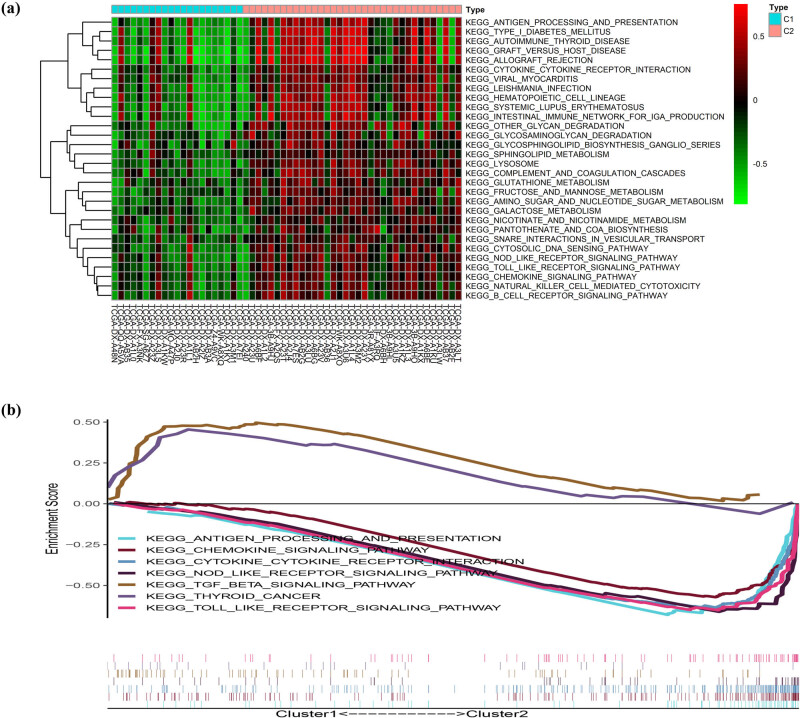
The differences in biological features in pyroptosis-associated clusters. (a) The results of a GSVA enrichment analysis. (b) The results of a GSEA.

We used the R package “ConsensusClusterPlus” to cluster TCGA DDL patients according to the expression level of 52 PRG quantities and ultimately identified 2 clusters. Cluster 1 consisted of 21 samples, while cluster 2 was composed of 35 samples, respectively (Figure 3a). Subsequently, a survival analysis was performed and showed that the prognosis of cluster 2 was remarkably better than cluster 1 (Figure 3b). Ten PRGS (NOD2, NLRC4, IL18, NLRP3, GSDMA, CASP5, AIM2, GZMB, IL1B, IL6), expressed notably different between two clusters, were all upregulated in cluster 2 (Figure 3c). To explore the differences in biological behavior between two clusters, a GSVA enrichment analysis was performed. The results revealed (Figure 4a) that all pathways were enriched in cluster 2 and some were associated with immune activation, including antigen processing and presentation, B-cell receptor signaling pathway, cytokine–cytokine receptor interaction, NOD-like receptor signaling pathway, and Toll-like receptor signaling. Moreover, considerable differences in these pathways were confirmed by GSEA (NOM *p*-value < 0.05, |NES| > 1). However, the transforming growth factor beta signaling pathway and thyroid cancer were enriched in cluster 1 (Figure 4b).

### 3.4 Characteristics of the tumor microenvironment (TME) in distinct clusters

**Figure 5:**
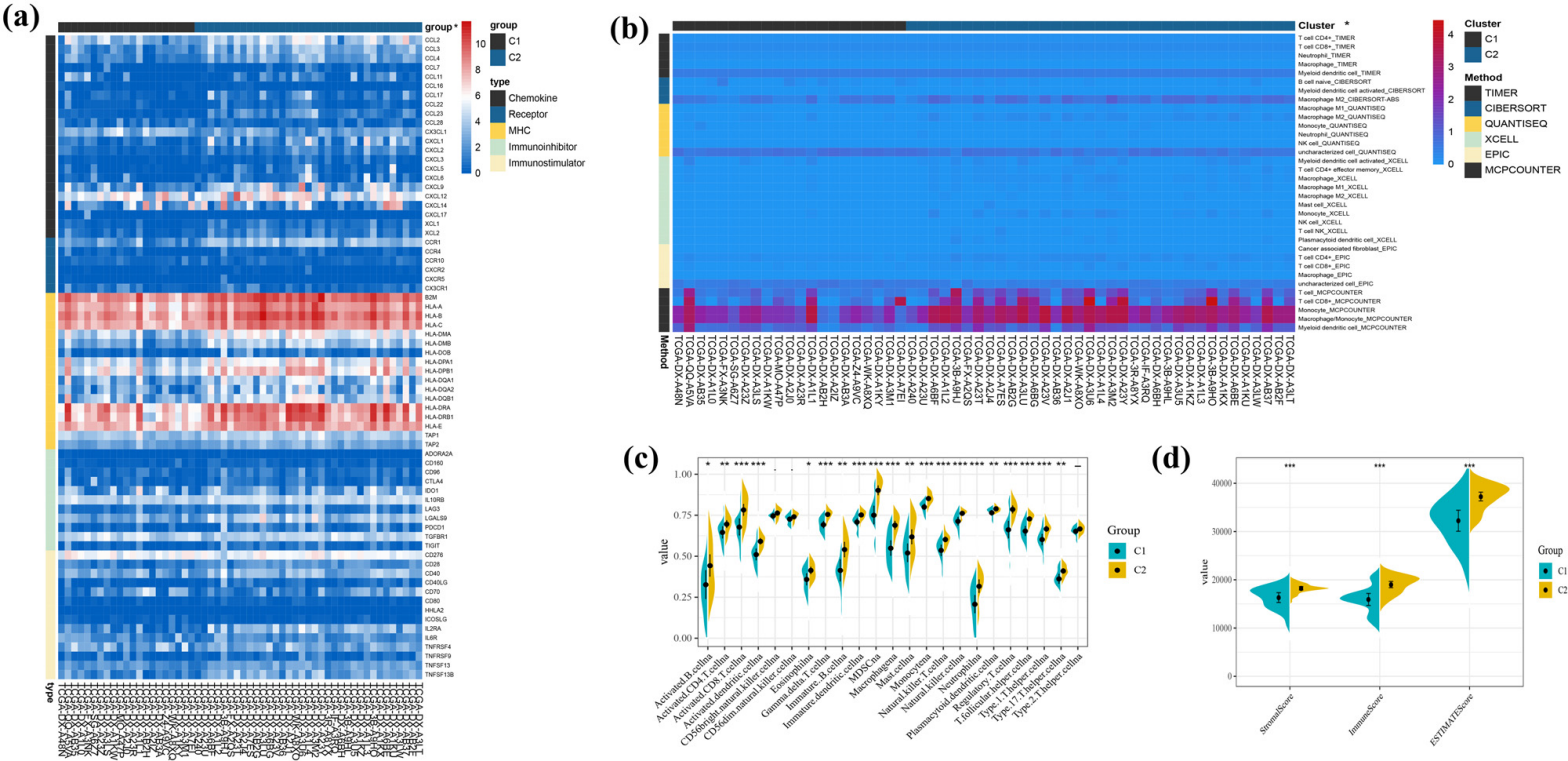
Variations in immune-related genes and the infiltration characteristics of TME cells in the pyroptosis-related clusters. (a) The thermogram shows variations in mRNA expression of chemokines, interleukins, alterons, and other cytokines among the two clusters. *p < 0.05. (b) The thermogram shows the frequency of TME-infiltrating cells among the two clusters. *p < 0.05. (c) The results of ssGSEA scores among the two clusters. *p < 0.05, **p < 0.01, ***p < 0.001. (d) Correlations between the two clusters and TME score. ***p < 0.001.

The results of GSVA and GSEA all suggest that PRG-related clusters may have a different immune status in DDLs. We first explored the various immune markers’ expression in different clusters, which included immune stimulatory or inhibitory genes, chemokine, chemokine receptors, and MHC genes. We found that 70 of 122 immune markers expressed differently in subcluster (Figure S1). In Figure 5a, we showed the significantly expressed immune markers, and most of MHC complex and part of chemokines and chemokine receptors were more highly expressed in cluster 2, including CXCL6, CXCL14, and CXCL12, which attracted dendritic cells (DCs) and CD8+ T cells and promote immune cell migration. Next, we used Timer, Cibersort, Quantiseq, Xcell, Epic, and Mcp_counter to calculate the level of immune cell infiltration in DDL samples. Additionally, more infiltration of T cells, cytotoxic lymphocytes, neutrophil, and macrophage was found in cluster 2 (Figure 5b).

We further applied ssGSEA to make a comparison of the enrichment scores of immune cells between subclusters. Through ssGSEA, we have observed significant differences in the infiltration of most immune cells between cluster 1 and cluster 2, except for NK cells and Th2 cells. The level of immune cells’ infiltration in cluster 2 was significantly higher than in cluster 1 (Figure 5c). Otherwise, we evaluated the TME score of each cluster by using the ESTIMATE package. Just as Figure 5d shows, the StromalScore, ImmuneScore, and ESTIMATEScore of cluster 2 were significantly higher than cluster 1.

**Figure 6:**
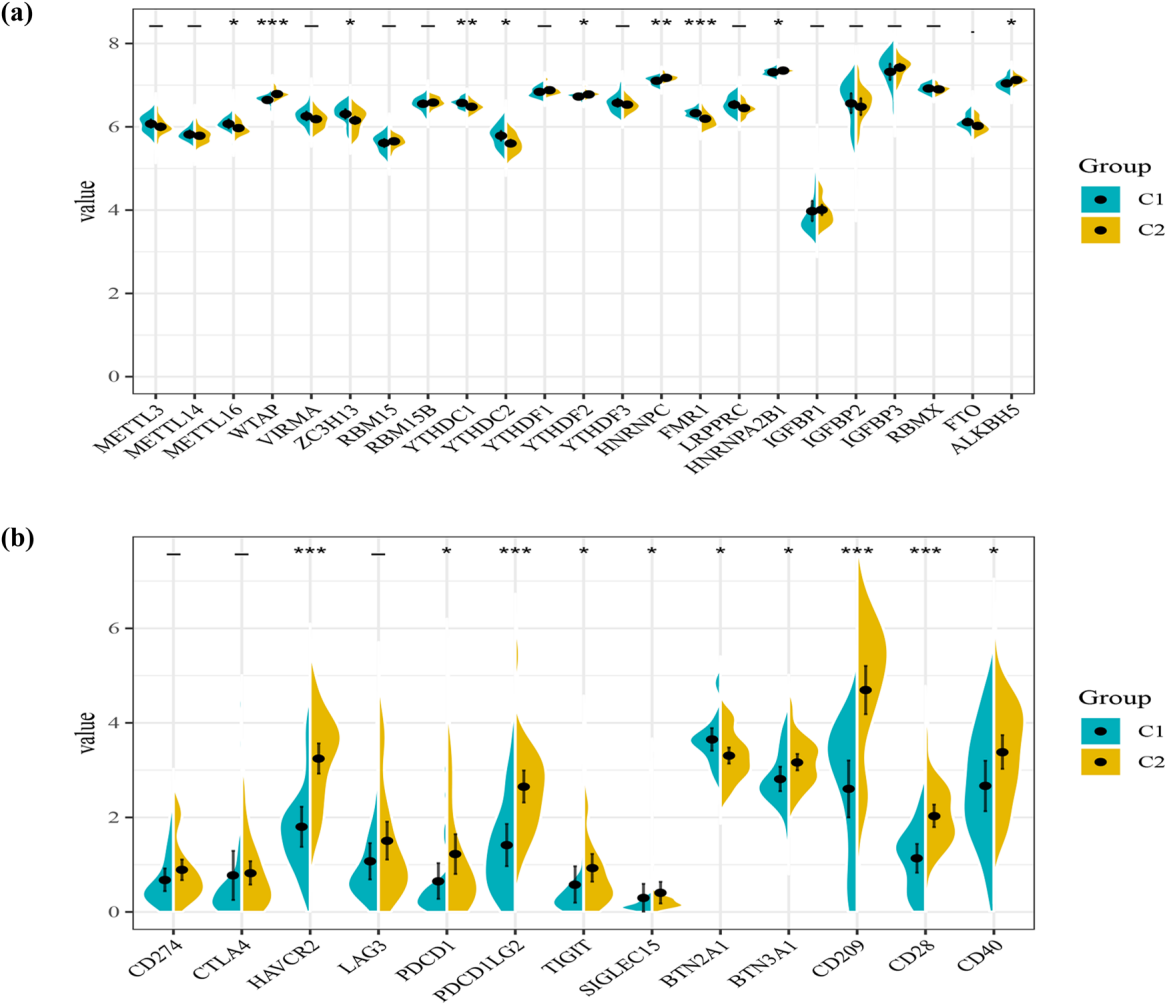
The relationship between clusters and m6A regulators and immune checkpoints. (a) Differences in expression of m6A regulators among the two clusters. (b) Differences in expression of immune checkpoint blockade genes among the two clusters. *p < 0.05, **p < 0.01, ***p < 0.001.

As one of the most common RNA modifications, M6A plays a role in regulating the initiation and progression of cancer. We also analyzed the expression levels of m6A regulatory genes in different clusters (Figure 6a). More expression of METTL16, ZC3H13, YTHDC1, YTHDC2, and FMR1 was found in cluster 1, while higher expression levels of WTAP, YTHDF2, HNRNPC, HNRNPA2B1, and ALKBH5 were found in cluster 2. With the use of checkpoint inhibitors, Immunotherapy is in full swing nowadays, prolonging patients’ survival in many types of tumors. We found immune checkpoint genes were expressed impressively different in clusters, including HAVCR2 (*p* < 0.001), PDCD1 (*p* < 0.05), PDCD1LG2 (*p* < 0.001), TIGIT (*p* < 0.05), SIGLEC15 (*p* < 0.05), BTN2A1 (*p* < 0.05), BTN3A2 (*p* < 0.05), CD209 (*p* < 0.001), CD28 (*p* < 0.001), and CD40 (*p* < 0.05) (Figure 6b), which indicated that these two clusters may respond differently to immune checkpoint inhibitors.

### 3.5 Development of a prognostic gene model based on pyroptosis-related clusters

**Figure 7:**
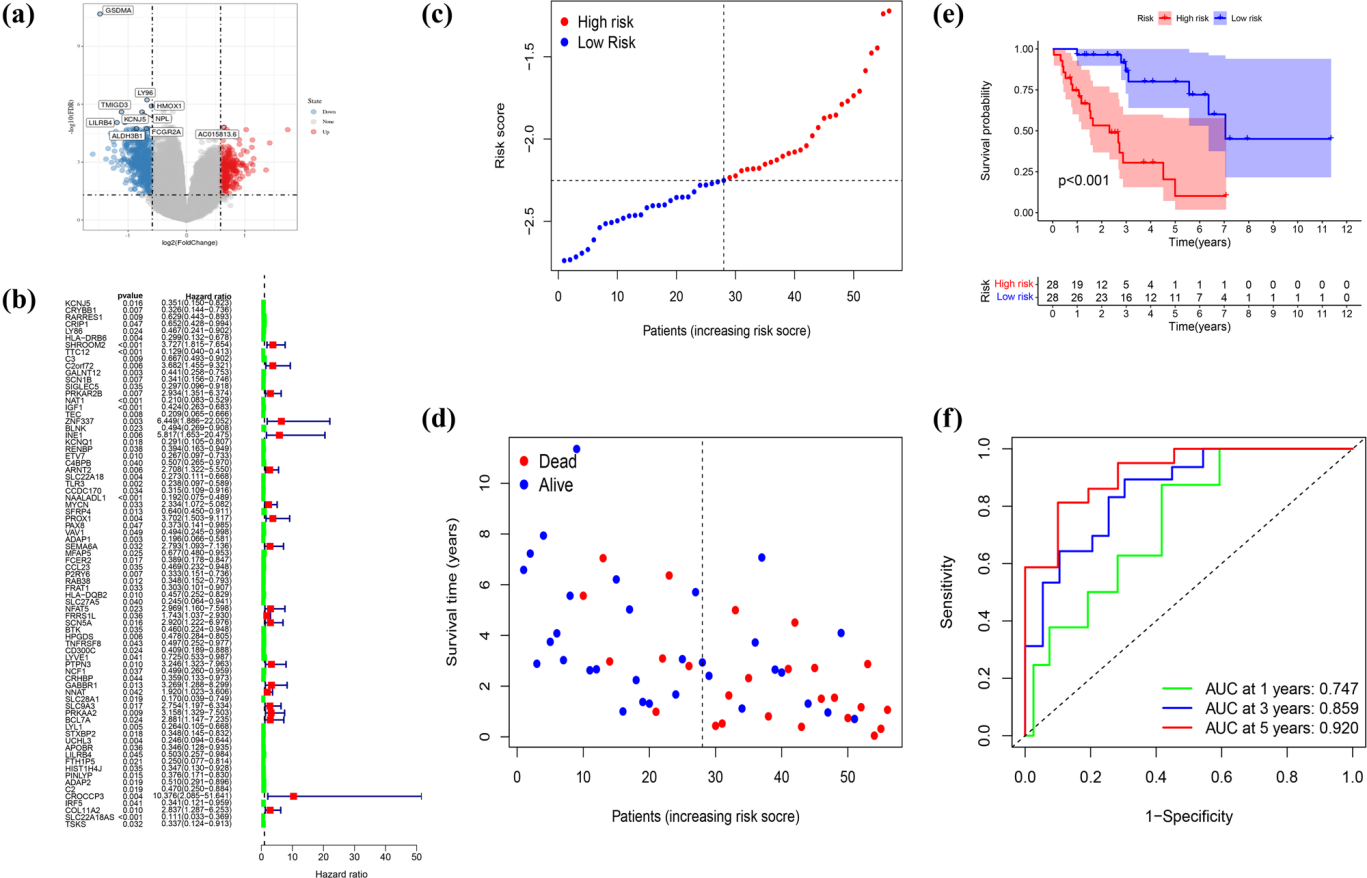
Generation of a gene expression signature to predict patient survival based on pyroptosis-related clusters in the TCGA cohort. (a) An overview of the differential gene expression between the two pyroptosis-related clusters in TCGA cohorts. (b) Univariate Cox regression analysis of OS in TCGA cohorts. (c) Distribution of patients based on the risk score. (d) The survival status for each patient (low-risk population: on the left side of the dotted line; high-risk population: on the right side of the dotted line). (e) Kaplan–Meier curves for the OS of patients in the high- and low-risk groups. (f) ROC curves demonstrated the predictive efficiency of the risk score.

**Figure 8:**
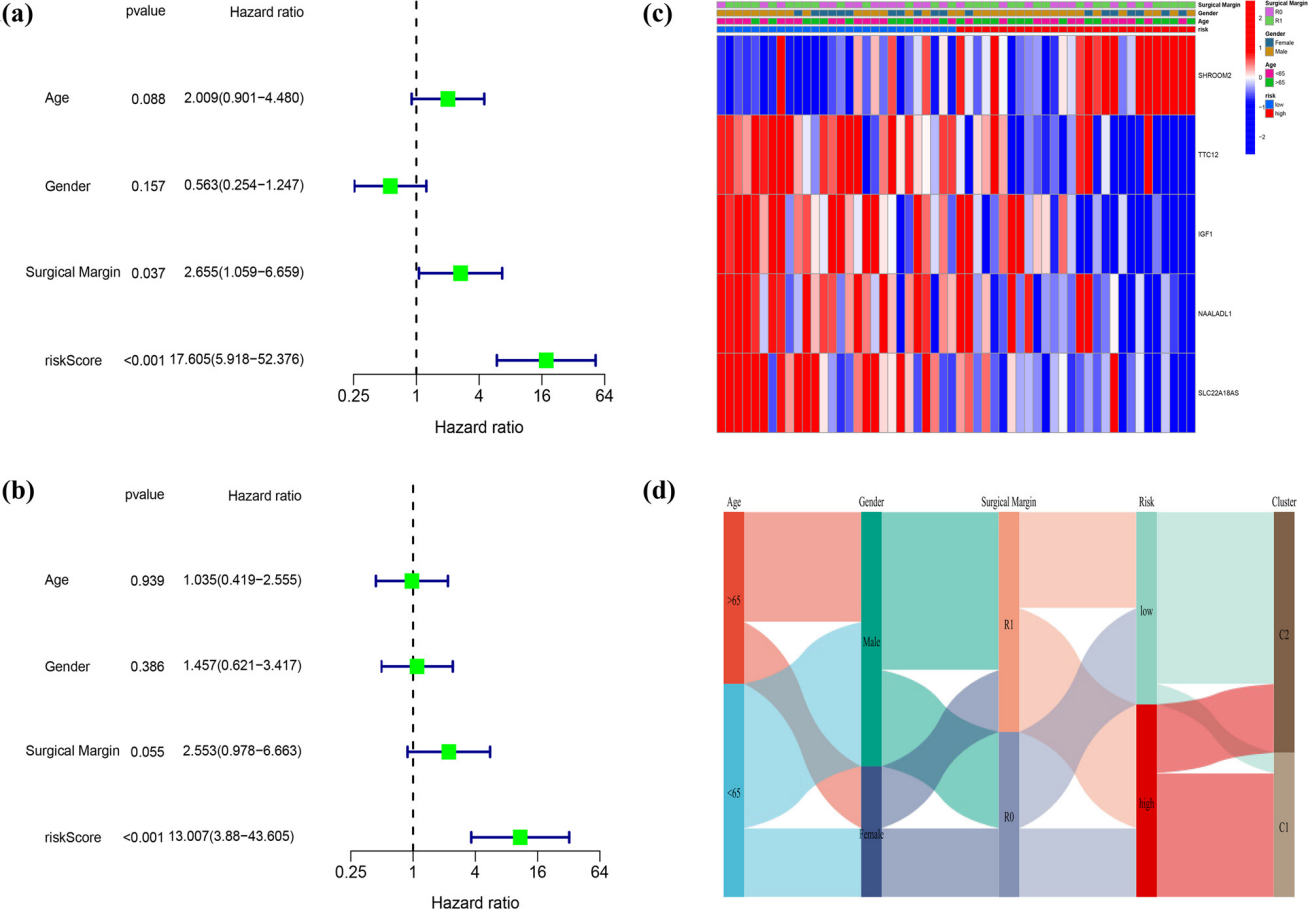
Univariate and multivariate Cox regression analyses for the risk score. (a) Univariate analyses for the TCGA cohort. (b) Multivariate analysis for the TCGA cohort. (c) Heatmap for the connections between clinicopathologic features and the risk groups. (d) Alluvial diagram showing the changes in the age, gender, surgical margin, risk group, and pyroptosis-related clusters.

For the better application of pyroptosis-related clusters in the treatment of DDL, we built a model that referred to the different genes between the two clusters next. A total of 1463 DEGs were identified, with an absolute value of |log2FC| ≥ 0.585 and *p* < 0.05 (Figure 7a). Seventy-seven genes were identified by univariate Cox regression analysis and all of those genes were regarded as survival-related genes (Figure 7b). By performing the LASSO-Cox regression analysis, a five-gene signature was constructed according to the optimum *λ* value (Figure S2a and b). We used these to build a risk score = (0.212 × SHROOM2) + (−0.403 × TTC12) + (−0.11 × IGF1) + (−0.027 × NAALADL1) + (−0.308 × SLC22A18AS). Then, 56 DDL patients were divided into low- and high-risk groups according to the median score (−2.25) (Figure 7c). The PCA and t-Distributed Stochastic Neighbor Embedding (t-SNE) showed that patients separated into two clusters showed different risks (Figure S2c and d). High-risk patients have a shorter survival time, just as Figure 7d and e proved. We applied time-dependent ROC analysis to evaluate the prognostic model’s specificity and sensitivity, and we found that the 1-year AUC was 0.747, 3-year AUC was 0.859, and the 5-year AUC was 0.920, respectively (Figure 7f). Then, we combined the risk score with age, gender, and Surgical Margin to stepwise Cox regression analysis. The results of univariate and multivariable Cox regression analyses consistently implied that risk score was an independent prognostic factor (Figure 8a and b). The alluvial diagram (Figure 8c) illustrated the distribution of patients in the two pyroptosis subclusters and two risk groups with clinical message. Most low-risk patients belonged to cluster 2 had a better prognosis. The decision curve analysis results and nomogram are shown in Figure S3.

### 3.6 External validation of the risk signature

**Figure 9:**
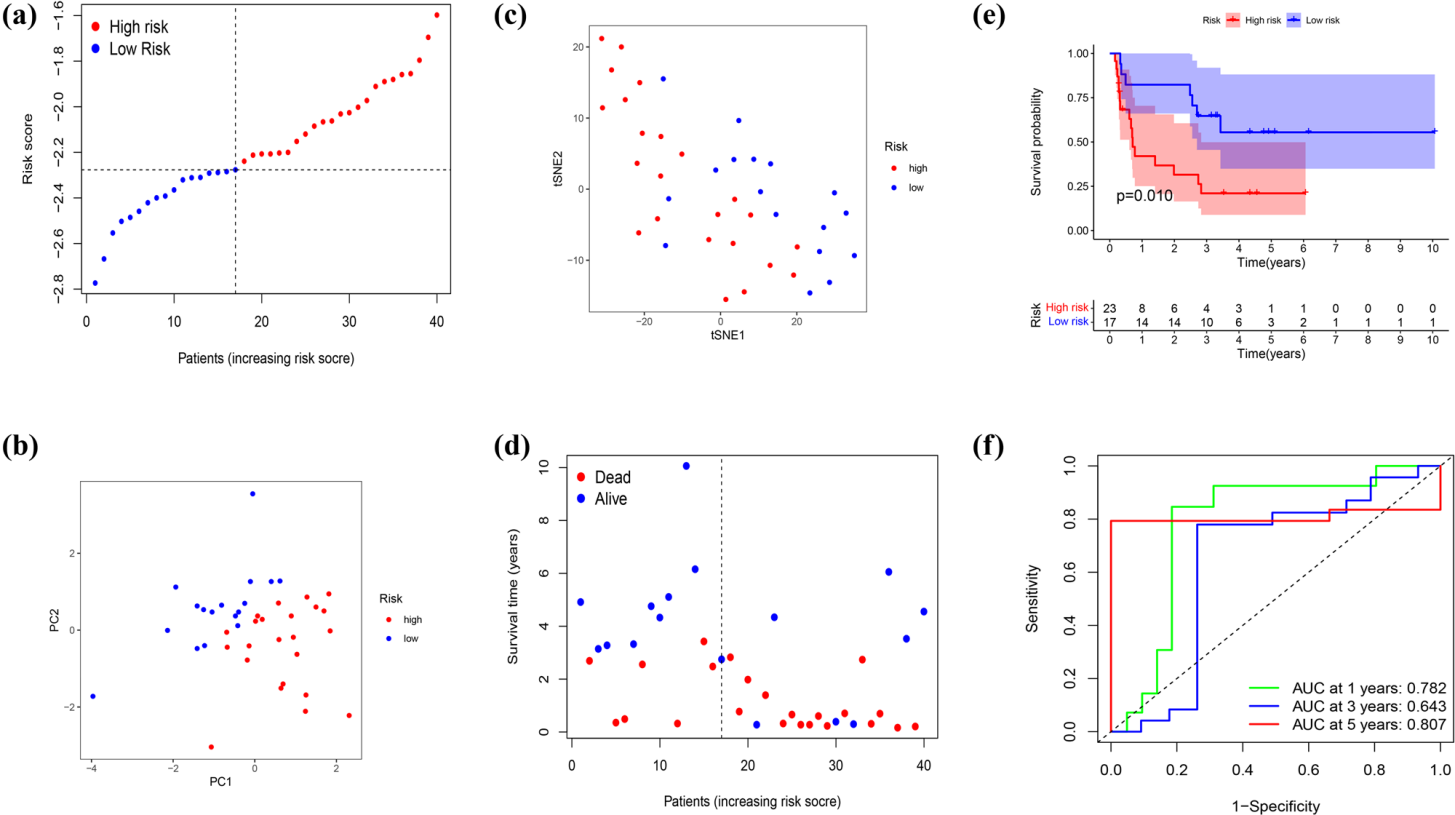
Validation of the risk model in the GEO cohort. (a) Distribution of patients in the GEO cohort based on the median risk score in the TCGA cohort. (b) PCA plot for DDL patients in the GEO cohort. (c) t-SNE plot for DDL patients in the GEO cohort based on the risk score. (d) The survival status for each patient (low-risk population: on the left side of the dotted line; high-risk population: on the right side of the dotted line). (e) Kaplan–Meier curves for comparison of the OS between the low- and high-risk groups. (f) Time-dependent ROC curves in the GEO cohort.

A validation cohort was set based on the data of 40 DDL patients from GEO database. Seventeen patients made up the low-risk group, and 23 patients composed the high-risk group, respectively (Figure 9a). Patients were well divided into two clusters through PCA and t-SNE analysis (Figure 9b and c), and patients in the low-risk group had a clear survival advantage over the high-risk group (Figure 9d). Moreover, Kaplan–Meier analysis also showed significant differences in the survival between the high-risk and low-risk groups (*p* = 0.01, Figure 9e). We further proved that the prognosis model was a good indicator for the 1-, 3-, and 5-year survival, and AUC was 0.782, 0.683, and 0.807 separately (Figure 9f).

### 3.7 Functional analyses of risk model

**Figure 10:**
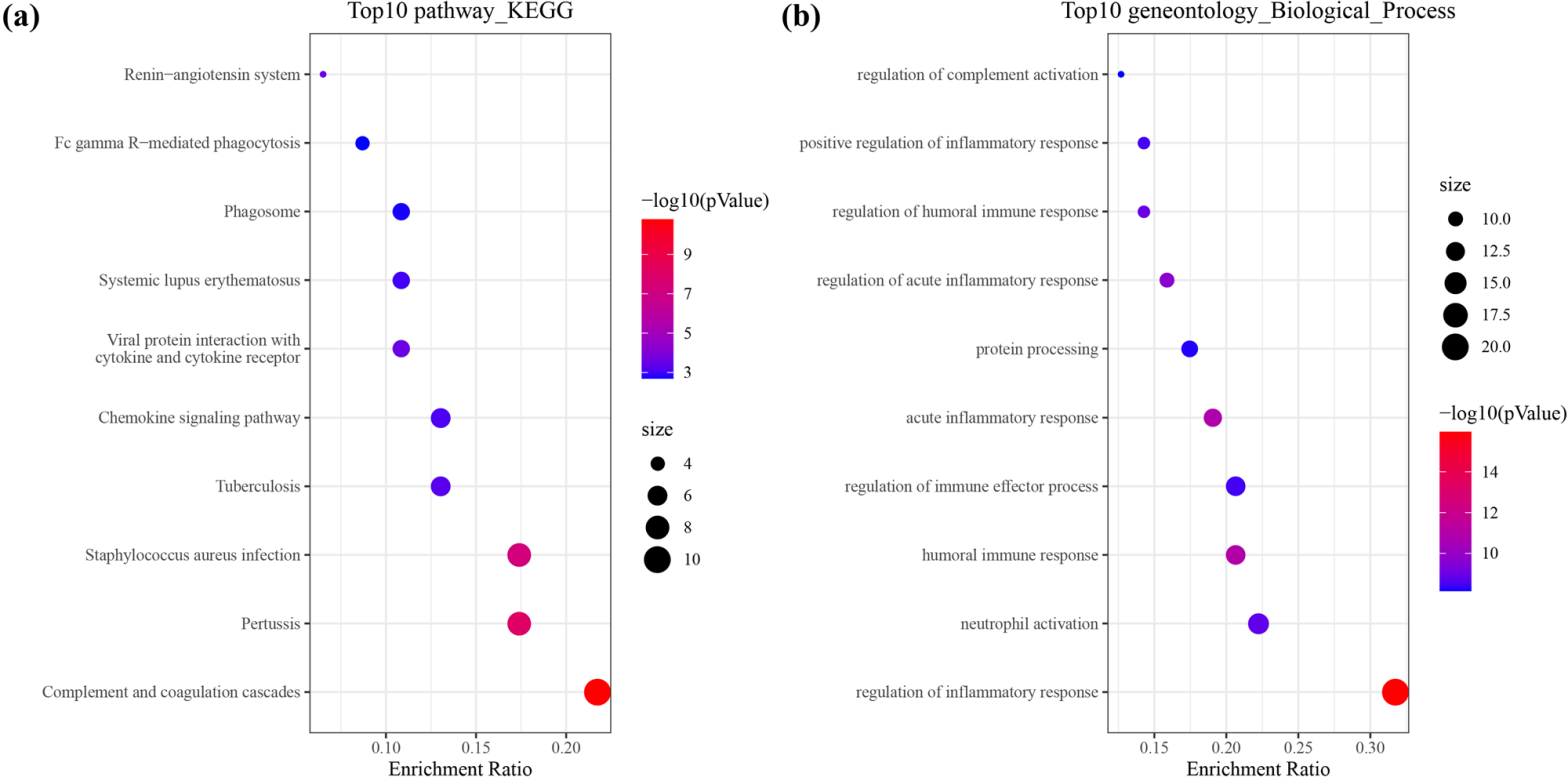
Functional analysis based on the DEGs between the two-risk groups in the TCGA cohort. (a) Bubble graph for KEGG pathways. (b) Bubble graph for GO-biological process enrichment. The bigger bubble means the more genes enriched, and the increasing depth of red means the differences were more obvious.

To explore the differences in pathways and gene functions between the high-risk and low-risk groups in the TCGA cohort, the “limma” R package was used, and finally, 67 DEGs were screened out. In the high-risk group, there were 9 genes upregulated and 58 genes downregulated. Complement and coagulation cascades, chemokine signaling pathway, and Fc gamma R-mediated phagocytosis were enriched in KEGG pathways (Figure 10a). The top five most enriched GO-biological process terms were as follows: regulation of inflammatory response, neutrophil activation, humoral immune response, humoral immune response, regulation of immune effector process, acute inflammatory response, and humoral immune response (Figure 10b). The results of GO-cellular component and molecular function are shown in Figure S4. These results indicated that the DEGs were mainly correlated with the immune response and inflammation.

### 3.8 Evaluation of the TME and immune activity between the high- and low-risk groups

**Figure 11:**
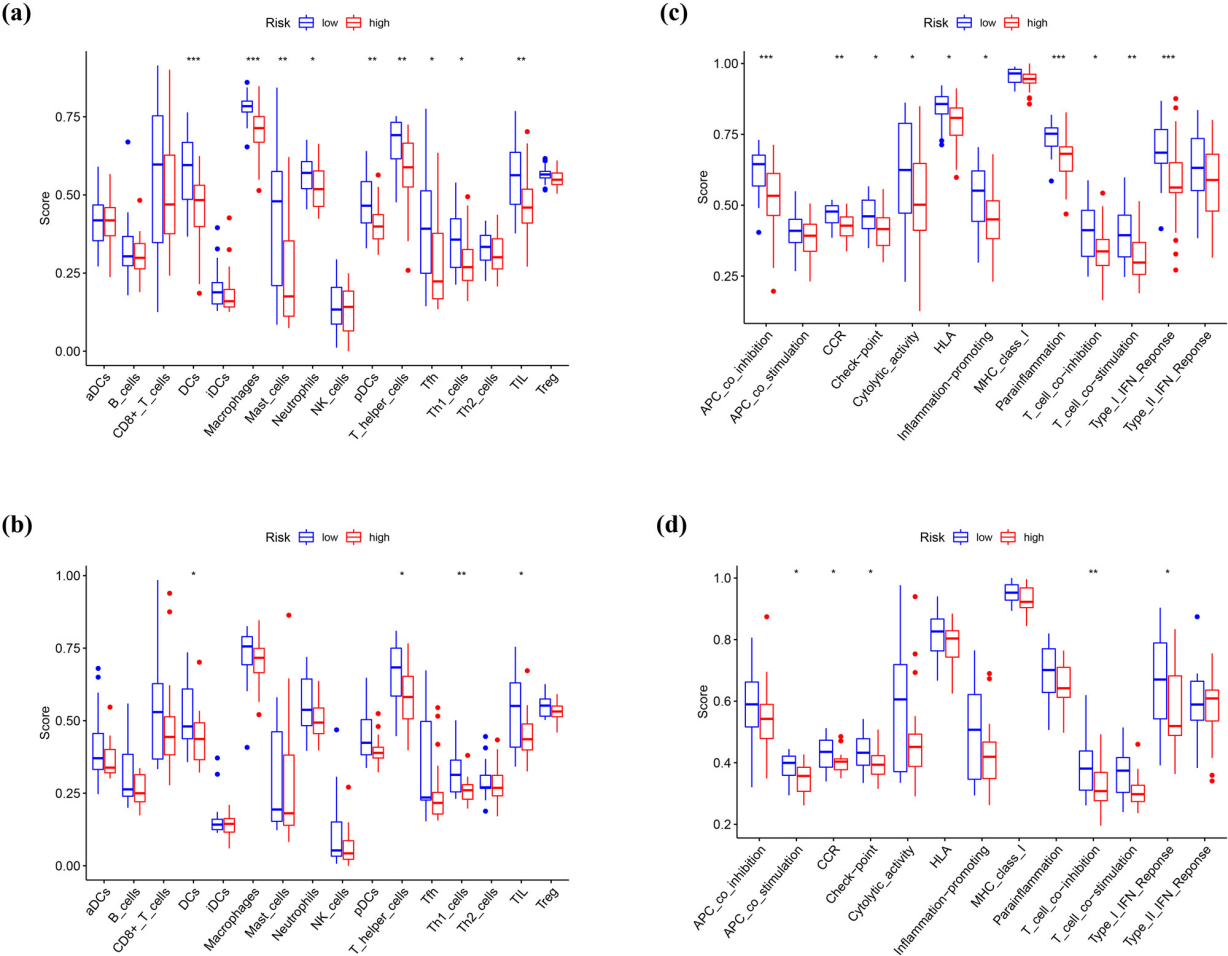
Comparison of the ssGSEA scores for immune cells and immune pathways. (a and c) Comparison of the enrichment scores of 16 types of immune cells and 13 immune-related pathways between the low- (blue box) and high-risk (red box) groups in the TCGA cohort. (b and d) Comparison of the tumor immunity between the low- (blue box) and high-risk (red box) groups in the GEO cohort. *p* values were shown as follows: ns, not significant; *p < 0.05; **p<0.01; ***p < 0.001.

Based on the KEGG and GO analyses, we can detect the different immune status in high- and low-risk groups. We first performed ESTIMATE to evaluate the immune environment. As shown in Figure S5, Immunesocre and ESTIMATEScore of the low-risk group were significantly higher than the high-risk group, no matter in TCGA or GEO cohorts. Then, the enrichment scores of 16 types of immune cells and the activity of 13 immune-related pathways were further compared by ssGSEA. We can observe most of the immune cells less infiltrated in the high-risk group in TCGA and GEO cohorts (Figure 11a and b), especially of DCs, T helper cells, T helper (Th) 1 cells (th1 cells), and tumor-infiltrating lymphocytes (TILs). Aside from APC co-stimulation, MHC-class-Itype-2, IFN response pathway, and the other 10 immune pathways showed lower activity in the high-risk group than in the low-risk group in the TCGA cohort (Figure 11c). The analysis of the GEO cohort disclosed similar results (Figure 11d). Though Timer, Cibersort, Quantiseq, Xcell, Epic, and Mcp_counter to evaluate the immune cell infiltration level between low-risk and high-risk groups, we found T cells, B cells, and macrophage cells were infiltrated more in low-risk samples in the TCGA cohort (Figure S6). All results together suggested that the high-risk group is in a state of immunosuppression.

## 4 Discussion

Inflammation is a recognized hallmark of cancer that substantially contributes to the tumorigenesis and progression of malignancies [32]. Pyroptosis, known as an inflammation-related way of programmed cell death, can be triggered by long-term exposure of tissues or cells to the inflammatory environment. Wang et al. [33] reported that alcohol accumulation could promote esophagitis through active pyroptosis and release of IL-18 and IL-1β in esophageal epithelial cells. Furthermore, caspase-1-derived pyroptosis was associated with esophageal cancer. Chen et al. [34] found that NEK7–NLRP3 interaction modulated the pyroptosis in inflammatory bowel disease (IBD). With increased expression in IBD, GSDMB can regulate epithelial repair [35]. As we know, IBD increases the risk of severe diseases like colon cancer [36]. However, pyroptosis plays a different role in cancers. It can be a tumor suppressor in colorectal cancer [17] and skin cancer [37]. lncRNA ADAMTS9-AS2 can inhibit GC cell viability by activating NLRP3-mediated pyroptosis [38]. Decreased GSDME is related to lymph node metastasis and worse prognosis in breast cancer [39], but GSDMB can be a tumor promoter that induces invasion and metastasis in breast cancer cells [40]. Just as those researchers have proven, pyroptosis acts as a “double-edged sword” in cancer so that we cannot evaluate the prognostic value of these gasdermins in tumors based on their expression alone.

DDL is the worst type of STS with aggressive clinical behavior, high recurrence rates, and the trend for metastasis. In recent decades, chemotherapy and target therapy have made progress in DDL. Anthracycline-based therapy is still the standard treatment for DDL [7,22,41], but recent literature suggests that eribulin [42], trabectedin [43], and pazopanib [44] may also benefit DDL patients. Since DDL highly expression of MDM2 and CDK4, the clinical trials about MDM2-targeted therapy and CDK4-targeted therapy have been conducted and demonstrated the clinical benefits and toxicity [45,46]. With the rise of immunotherapy, multicenter trials about immune-checkpoint inhibitors such as nivolumab and pembrolizumab were launched and exhibited clinical benefits [47,48]. However, the precise role of these agents remains to be elucidated, and the efficacy is still limited. The search for the new target remains imperative work for DDL patients.

TME can influence the tumor development and progression [49]. TILs were considered prognosis-related agents in numerous cancers [50]. Through the analysis of immune cell infiltration in the DDL group and the adipose group, we found that the proportion of T-cell CD8, T-cell CD4, NK cells activated, macrophages, and DC cells activated was lower in DDL tissues, which may indicate that DDL patients were in a state of immunosuppression. Emerging evidence unveils the possible association between pyroptosis and the tumor immune microenvironment [51,52]. We separated DDL patients into two completely different clusters on the ground of 52 PRGs. Our results show: (1) most PRGs showed high expression in cluster 2 and (2) cluster 2 showed a better prognosis. We also observed that expression of IL-6 and IL-18 was significantly increased in cluster 2, uncovering that cluster 2 exhibits a dramatic response of inflammatory, which is possibly mediated by pyroptosis. We further analyzed the differences of immune state in clusters. As we know, TME includes fibroblasts, immune cells, diffusible cytokines, and chemokines secreted from cancer. In our results, most of MHC complex, chemokines, and chemokine receptors were more highly expressed in cluster 2, T cells, cytotoxic lymphocytes, neutrophil, and macrophage were infiltrated more in cluster 2, and stromal score, immune score, and estimate score were higher in cluster 2. The cluster 2 was also characterized by a significant immune activation, including antigen processing and presentation, B-cell receptor signaling pathway, cytokine–cytokine receptor interaction, NOD-like receptor signaling pathway, and Toll-like receptor signaling pathways. Our study indicates that the higher infiltrating immune cells and stronger immune activation in DDL are related to a better prognosis, and pyroptosis levels were closely related to TME.

In the era of immunotherapy, positive expression of immune checkpoint molecular has been the criteria for the eligible patients. Patients with high expression of PD-L1 are generally more sensitive to immunotherapy [53]. Park et al. reported that 21.9% (7/32) of the DDL patients were PD-L1 positive [54]. Tawbi et al. also reported that the expression level of PD-L1 was increased dramatically in DDL compared with other types of STS [47]. These studies suggest the possibility of immunotherapy to treat advanced DDL. However, only a few DDL patients manifest effective response to immunotherapy, suggesting that additional strategies are needed to promote anti-tumor immunity. We found higher expression of immune checkpoint molecular in cluster 2, and pyroptosis can lead to activation of DCs and macrophages in tumor to better present tumor antigens, secrete cytokines and chemokines, enhancing T-cell recruitment and activation, so we speculate that the group of DDL patients with high expression PRGs may more fit the immunotherapy.

A wide variety of prognosis-correlated models have been built in multiple cancer types to facilitate tumor management and treatment. Considering the crucial role pyroptosis plays in the patients’ outcome and immune regulation of DDL, it is essential to establish a scoring system to evaluate the clinical outcome linked with pyroptosis in DDL patients. To make a better application in clinical works, we built a model to value the prognostic risk on the base of DEGs between the two pyroptosis-related clusters. Our study generated five signature featuring genes (SHROOM2, TTC12, IGF1, NAALADL1, SLC22A18AS) and found that they could provide benefits for clinical management and predict survival in DDL patients. IGF1, a member of the insulin superfamily, is an important regulator of tissue growth and development and is linked to the development of numerous cancers [55,56]. IGF/IGF1R was reported as an independent predictor of the malignant potential of adult STS [57]. SHROOM2 has been considered to be the carcinogenesis of colorectal cancer and esophageal squamous carcinoma [58,59]. Our risk model linked prognosis with pyroptosis but was not limited to PRGs. Moreover, this model represented DDL patients with various clinical traits and was linked to immunomodulation. The high-risk group presented a worse outcome. Chen et al. reported that positive surgical margin was an independent predictor of poor OS in primary retroperitoneal liposarcoma [60]. However, the results of other studies show that the surgical margin was only associated with DDL local recurrence-free survival, rather than the OS [61]. And our risk model casts better light on patient outcomes than surgical margins. Furthermore, immune infiltrated cells and TME score in the high-risk group were weightless, exhibiting a wide disorder of immune functions, which was verified by GEO datasets.

In our investigation, it has been observed that both cluster 1 identified through PRGs and the high-risk group established based on key genes exhibit pronounced immunosuppressive status and inferior prognostic outcomes. Numerous studies have already established a correlation between immunosuppression and adverse prognosis in tumors [62]. Patients with immunosuppressive tumor status typically demonstrate reduced immune cell infiltration, higher tumor grading and staging, elevated rates of recurrence and metastasis, as well as poorer survival rates [63]. Furthermore, immunosuppression can also impact the tumor’s sensitivity to immune therapies, diminishing its responsiveness to immunotherapeutic agents such as immune checkpoint inhibitors [64]. Understanding the influence of tumor immunosuppression on prognosis aids in guiding tumor treatment choices and optimization, with the aim of enhancing patient outcomes and survival rates [65,66]. For the high-risk group, strategies such as inhibiting immune suppressive factors, enhancing immune cell functionality, and rectifying immune evasion mutations can be employed to ameliorate the tumor’s immune response. As for the low-risk group identified by the model, exploration of immune therapies may potentially yield greater clinical benefits.

Overall, with multi-perspective and cross-validation of the database, our study classifies DDL patients into clusters, identifies DEGs, and builds a prognostic model, suggesting that pyroptosis is bound up with DDL. The development of our model enables the categorization of patients with DDL and facilitates targeted therapeutic interventions. Through the discovery of novel biomarkers, this model assists in the diagnosis, prognostic evaluation, and therapeutic monitoring of diseases, leading to the provision of more accurate and reliable clinical information. However, our study has several limitations that are worth considering. First, all data are from public databases, further research *in vitro* and *in vivo* experiments are needed for better evaluation. Second, data on some important clinical variables such as chemotherapy and immunotherapy were not available in our database. Our study is the first bioinformatics study dedicated to pyroptosis in DDL, and our findings emphasized the crucial clinical implications of pyroptosis-related genes and provided new strategies for personalized therapy, including immunotherapy, in patients with DDL.

## Supplementary Material

Supplementary Figure
