# Supplementary material for: The pyroptosis-related signature predicts prognosis and influences the tumor immune microenvironment in dedifferentiated liposarcoma
Source: Open Med (Wars). 2024 Jan 9;19(1):20230886. doi: 10.1515/med-2023-0886 (PMC10787309; doi:10.1515/med-2023-0886)
Supplement: Supplementary Figure [file med-2023-0886-sm.pdf]

Supplementary material

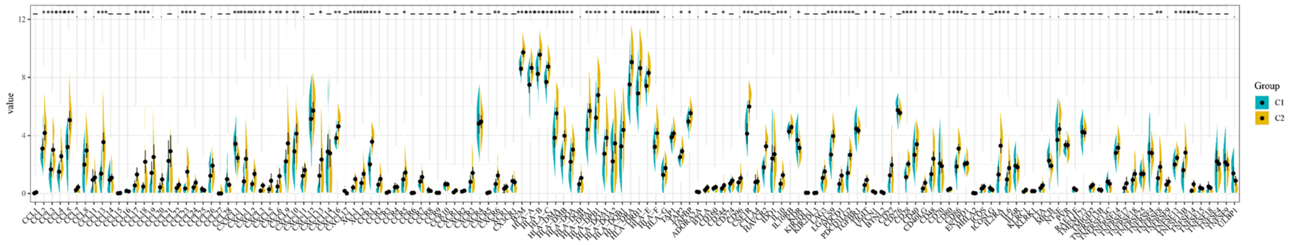

Figure S1: The different expression of immune markers in pyroptosis related clusters. *$P < 0.05$, **$P < 0.01$, ***$P < 0.001$.

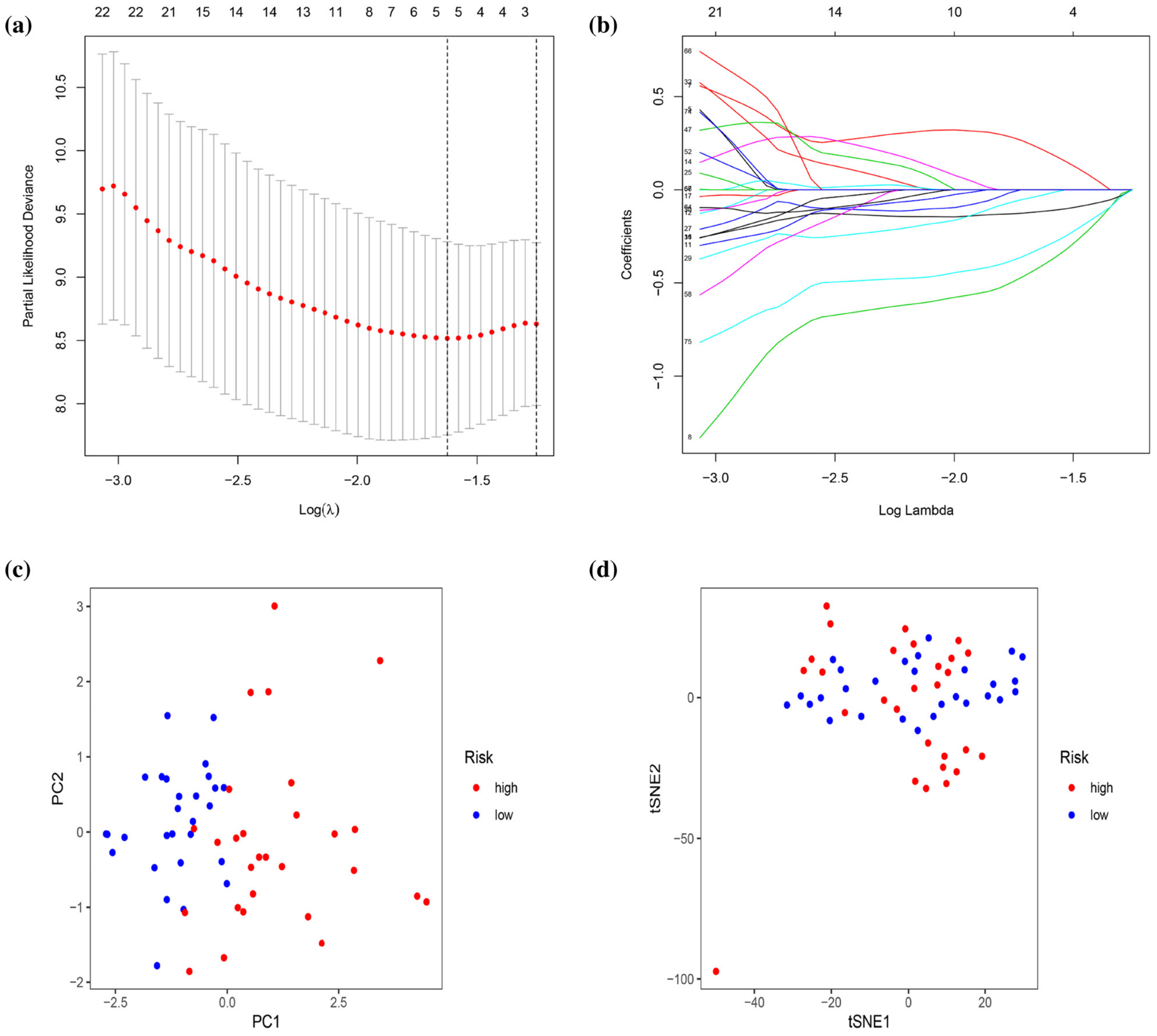

Figure S2: Construction of risk signature in the TCGA cohort. (a) LASSO regression of the 5 OS-related genes. (b) Cross-validation for tuning the parameter selection in the LASSO regression. (c) PCA plot for DDL patients based on the risk score. (d) t-SNE plot for DDL patients based on the risk score.

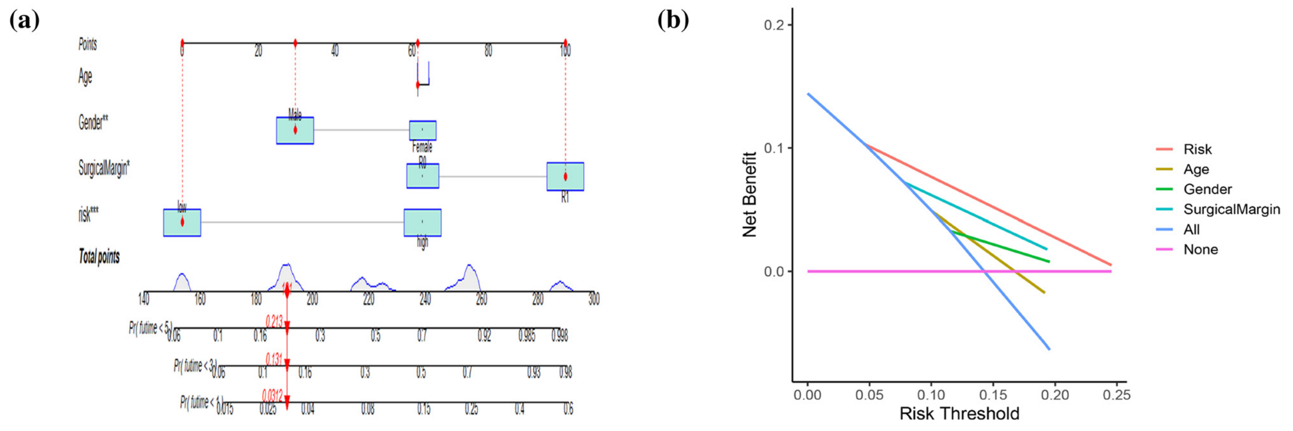

Figure S3: The prognostic signature base on the clusters-related DEGs for DDL in TCGA cohort. (a) Nomogram for predicting the probability of 1-, 3-, and 5-year overall survival time for DDL. (b) DCA results.

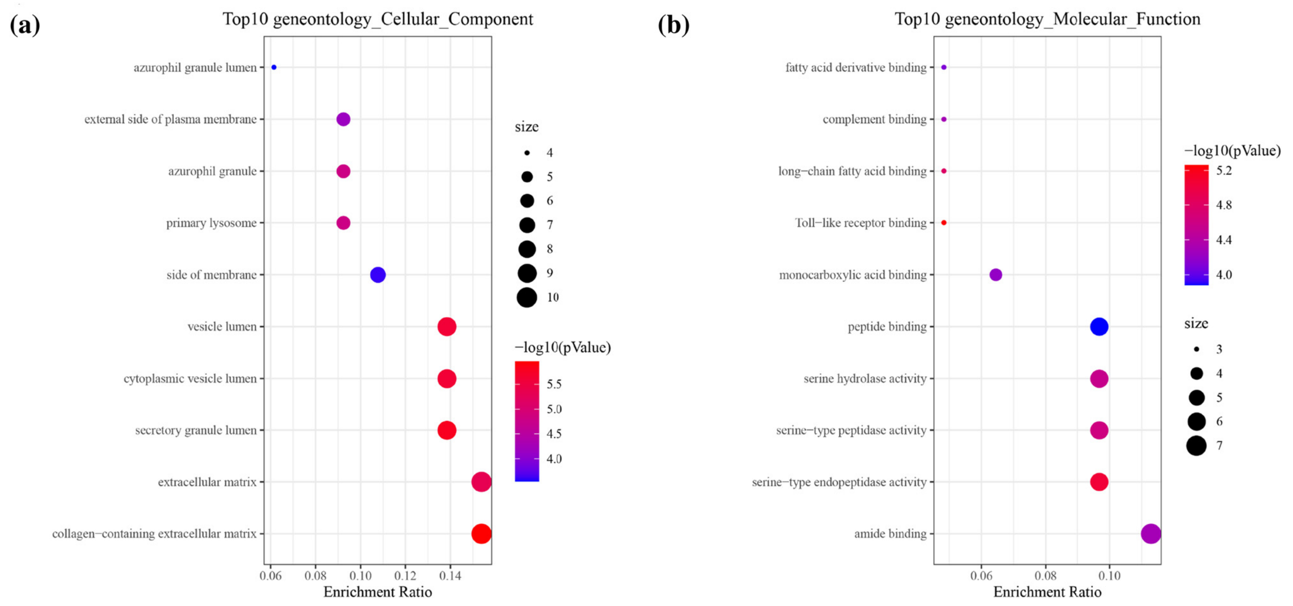

Figure S4: The results of GO- cellular component and molecular function enrichment based on the DEGs between the two-risk groups in the TCGA cohort.

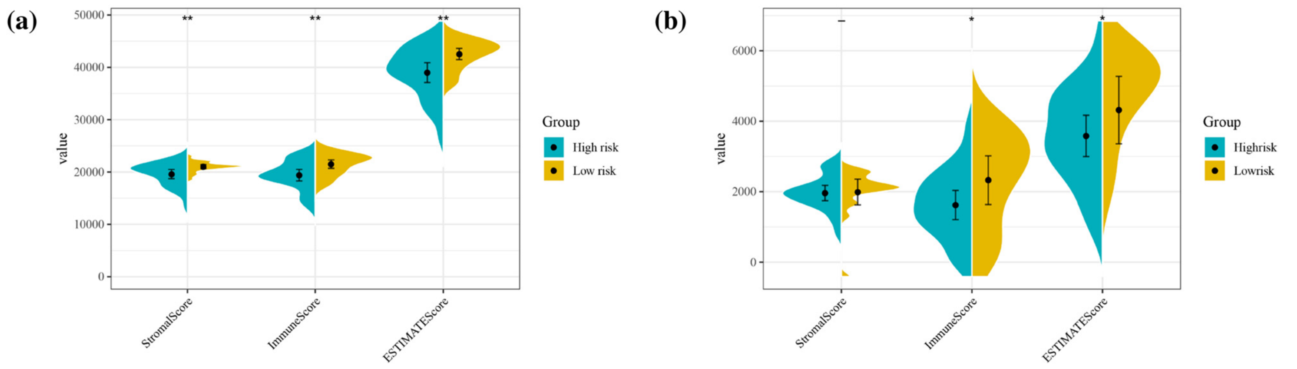

Figure S5: Evaluation of the TME between the two risk groups. (a) Correlations between risk group and TME score in TCGA cohort. (b) Correlations between risk group and TME score in GEO cohort.

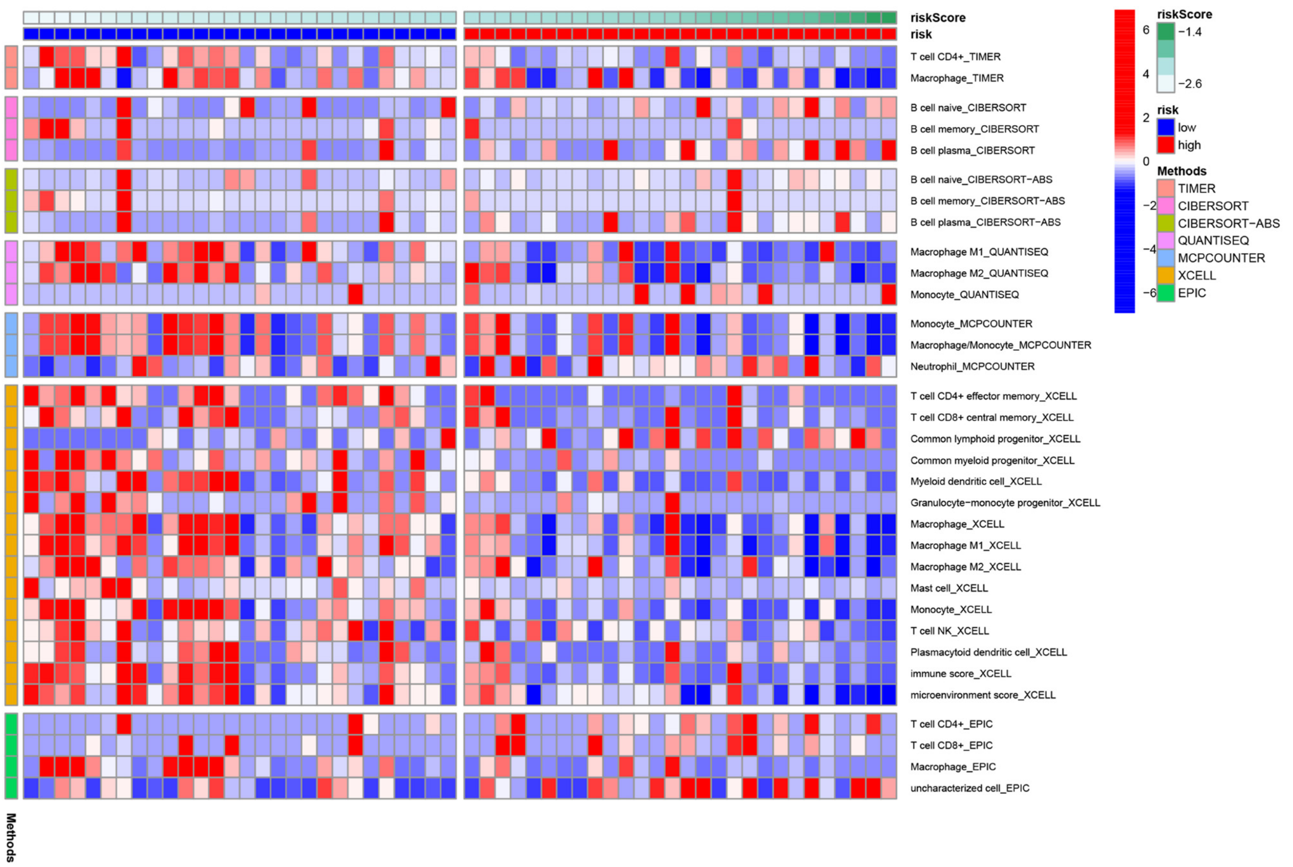

Figure S6: The thermogram shows the frequency of TME infiltrating cells and immune score among the risk groups.
